# Supplementary material for: Deep brain stimulation-guided optogenetic rescue of parkinsonian symptoms
Source: Nat Commun. 2020 May 13;11:2388. doi: 10.1038/s41467-020-16046-6 (PMC7220902; doi:10.1038/s41467-020-16046-6)
Supplement: Supplementary file 1 — Supplementary Information [file 41467_2020_16046_MOESM1_ESM.pdf]

Supplementary Information for

Deep brain stimulation-guided optogenetic rescue of parkinsonian symptoms

Valverde *et al*.

Correspondence to: laurent.venance@college-de-france.fr

This PDF file includes:

Supplementary Figures and legends S1 to S7

Supplementary Tables S1 to S3

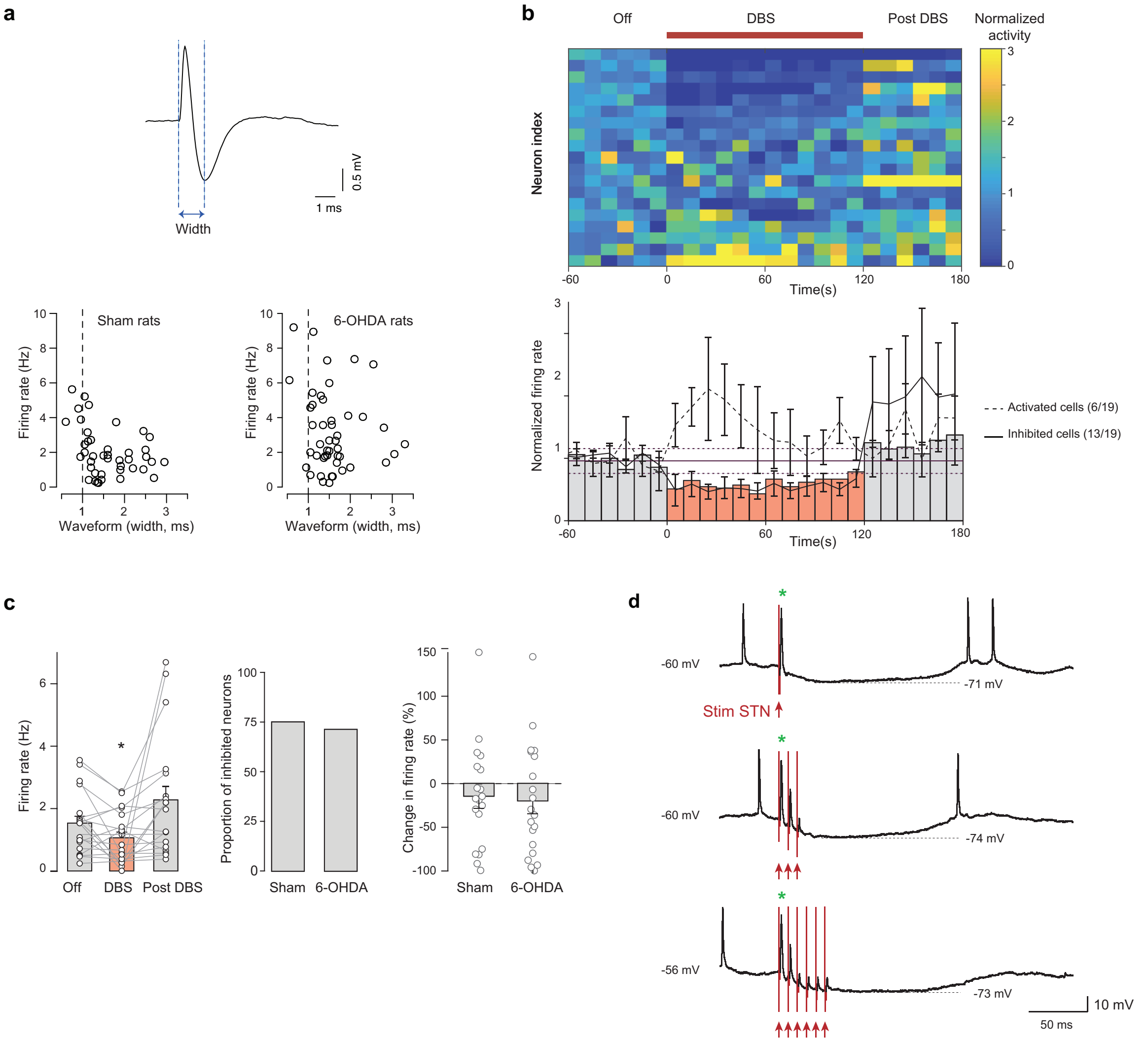

Supplementary Figure 1. ***In vivo*** **DBS reduces M1 pyramidal cells activity in rats.**
a, Representative trace of a single action potential of a putative pyramidal neuron recorded in the rat orofacial motor cortex. Dashed lines delineate waveform width, from start to negative trough. Plot indicates mean spontaneous firing rate against waveform duration for all recorded neurons in Sham and 6-OHDA rats. Neurons with broad waveforms (> 1 ms) were considered as putative pyramidal neurons, the rest were excluded from analysis. **b**, Heatmap of individual cortical neurons normalized firing rate (top), and averaged time course (bottom), before, during and after DBS (10s bins) in sham rats: bars correspond to the median of all neurons (mean±2SD of the baseline is indicated) and separate time courses (mean±SEM) for activated and inhibited neurons are superimposed. Under DBS, 68% of the pyramidal cells were inhibited while 32% were activated (n=19). **c**, DBS decreases cortical activity in sham rats (p=0.0266, paired t-test, n=19 neurons) (mean±SEM and individual neurons are represented). The proportion of inhibited neurons and the magnitude of the effect on the firing rate are similar in sham- and 6-OHDA-lesioned rats (respectively: p=1, Fisher's exact test, and p=0.7894, t-test; n=19 neurons in sham rats, and n=20 in 6-OHDA-lesioned rats); for the magnitude of the effect, mean±SEM and individual neurons are represented. **d**, Example of an intracellularly recorded M1 pyramidal cell antidromically activated by STN stimulation (red arrow: STN stimulation; green star: antidromically evoked spike). These cells (n=4) also displayed a pause in firing after the antidromic spike, associated with an hyperpolarization of the membrane potential (upper panel). Upon repetitive STN stimulations at 130 Hz (middle and lower panels), the antidromic spike amplitude is dramatically reduced and the amplitude of the hyperpolarization is increased. All statistical tests are two-tailed. *: p<0.05.

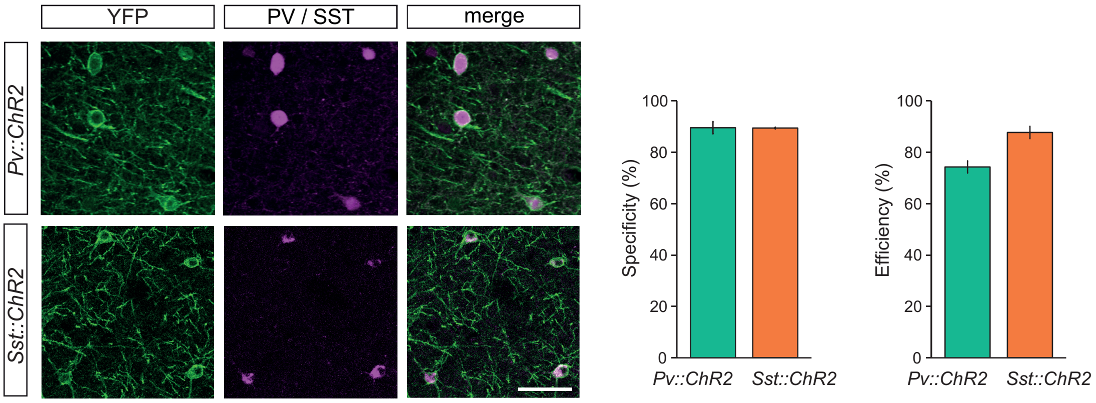

Supplementary Figure 2. Specific ChR2 expression in PV and SST cortical interneurons. Representative confocal pictures of M1 cortex in *Pv::ChR2* (top) and *Sst::ChR2* (bottom) mice, with double immunostaining for YFP (ChR2 expression reporter, green), and either PV (for *Pv::ChR2* mice) or SST (for *Sst::ChR2*) (pink) (right panels). Quantification of the specificity (number of double labeled cells / number of YFP-positive cells) and efficiency (number of double-labeled cells / number of PV- or SST-positive cells) of ChR2 expression in *Pv::ChR2* and *Sst::ChR2* mice (mean±SEM of 4 hemispheres from 2 mice in each mouse strain) (left panels). Scale bar: 50 µm.

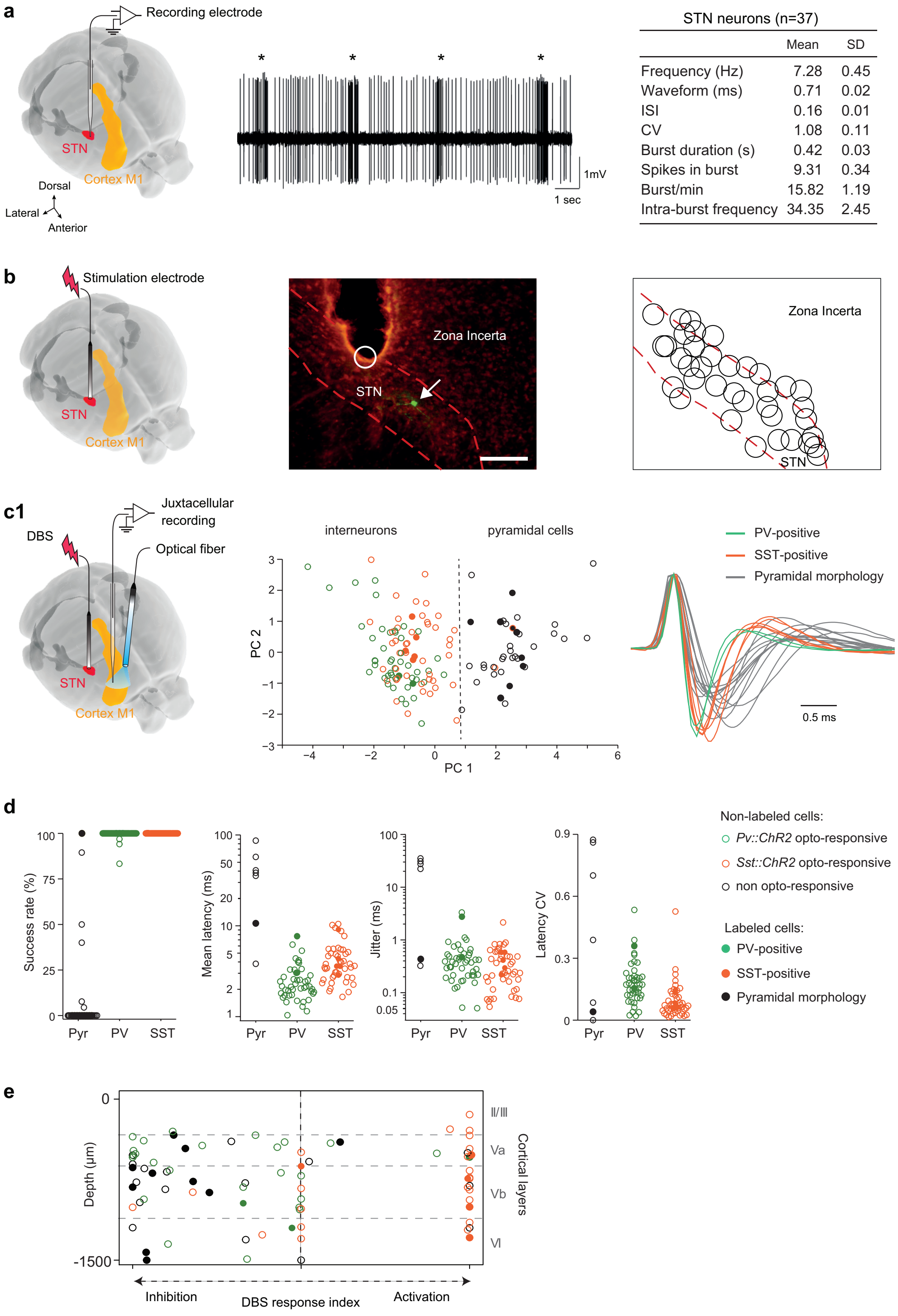

Supplementary Figure 3. Methodology for STN targeting and identification of M1 neurons in mice.

a, Electrophysiological identification of STN position. Left, a microelectrode is lowered in a stereotaxically defined region until encountering a neuron with typical STN firing patterns. Middle, extracellular recording of an STN neuron (stars indicate bursts as detected by the Poisson surprise method). Right, table represents the electrophysiological characteristics of STN neurons (n=37). **b**, The DBS stimulation electrode is lowered in the same coordinates as

the identified STN neurons. Middle, photomicrograph shows a juxtacellularly labeled STN neuron (white arrow) in close proximity to the tip of a stimulating electrode (white circle) (n=3 independent experiments with similar results). Scale bar=250µm. Right, schematic representation of the location of all electrically induced lesions of the STN. All data obtained with lesions outside of the STN were not included in the study. **c**, With the DBS electrode in place, an optical fiber is placed on top of the M1 cortex and a microelectrode is lowered close to the optical fiber until encountering a photo-responsive neuron. Middle, principal component analysis of waveform characteristics and opto-response properties reveal cutoff boundaries for discriminating pyramidal neurons (n=41) from interneurons (n=94). Two opto-responsive neurons with non-matching morphology and waveform were excluded. Right, waveforms of all labeled neurons (SST in orange, PV in green and pyramidal neuron in grey. **d**, Success rate, mean latency, jitter and latency CV of the response to opto-activation in all neurons (pyramidal neurons in black, n=41, PV in green, n=46 and SST in red, n=48; immunohistochemically or morphologically identified neurons are represented as filled circles). **e**, Cortical depth of all recorded neurons (based on position of labeled neurons or on manipulator depth) plotted according to their response to DBS (n=72 neurons).

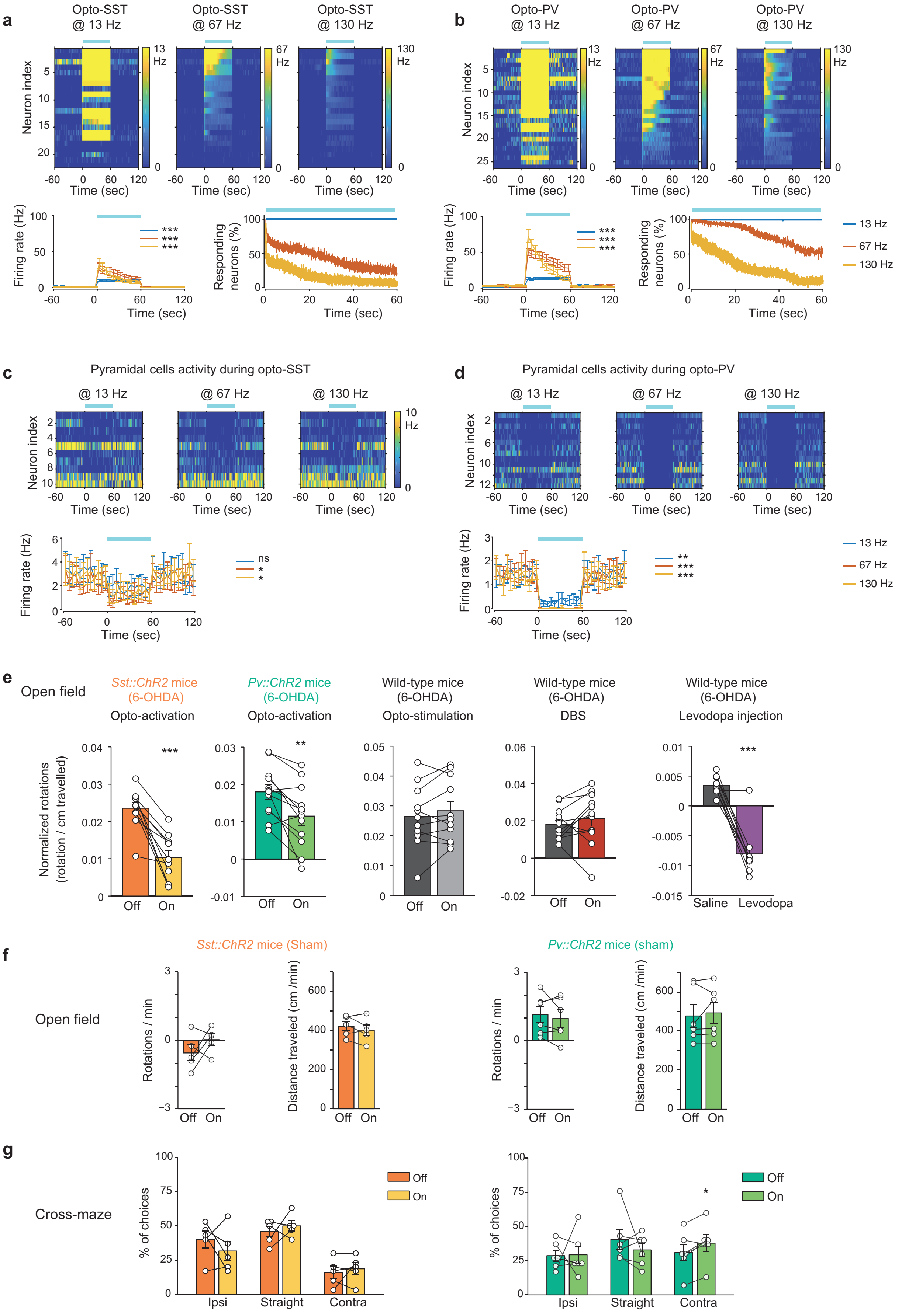
a
Opto-SST @ 13 Hz
Opto-SST @ 67 Hz
Opto-SST @ 130 Hz
Neuron index
Time (sec)
Firing rate (Hz)
Responding neurons (%)
b
Opto-PV @ 13 Hz
Opto-PV @ 67 Hz
Opto-PV @ 130 Hz
13 Hz
67 Hz
130 Hz
c
Pyramidal cells activity during opto-SST
@ 13 Hz
@ 67 Hz
@ 130 Hz
ns
d
Pyramidal cells activity during opto-PV
e
Open field
Sst::ChR2 mice (6-OHDA) Opto-activation
Pv::ChR2 mice (6-OHDA) Opto-activation
Wild-type mice (6-OHDA) Opto-stimulation
Wild-type mice (6-OHDA) DBS
Wild-type mice (6-OHDA) Levodopa injection
Normalized rotations (rotation / cm travelled)
Off
On
Saline
Levodopa
f
Sst::ChR2 mice (Sham)
Pv::ChR2 mice (sham)
Open field
Rotations / min
Distance traveled (cm /min)
g
Cross-maze
% of choices
Ipsi
Straight
Contra

Supplementary Figure 4. Additional results related to Figure 5.
A and **b**, Increased activity in SST (n=22) (**a**) and PV (n=26) (**b**) interneurons in response to opto-activation at 13, 67 and 130Hz (SST: 13Hz: p=9.2x10^{-5}; 67Hz: p=4.0x10^{-5}; 130Hz: p=6.1x10^{-5}; PV at all frequencies: p=8.3x10^{-6}; Wilcoxon's signed rank test); Top: heatmap of individual neuron activity at each frequency; Bottom left: averaged time course (mean±SEM). Bottom right: for each opto-pulse, proportion of SST and PV responding by at least 1 spike (among neurons responding to 3ms pulses: n=11 out of 22 SST cells, n=17 out of 26 PV cells). At 13Hz, all neurons fired at each pulse (SST and PV). At 130Hz, the success rate decreased along time and only 7% of SST and 10% of PV responded (last 10s), with 45% of the SST and 18% of the PV being silenced (<1% success rate/pulse). At 67Hz, 26% of SST and 55% of PV were responding at a given light pulse, with 18% of the SST and 0% of the PV being silenced (last 10s). **c** and **d**, Inhibition of pyramidal cells to opto-SST (**c**) or opto-PV (**d**). Top: heatmap of individual neuron activity. Bottom: averaged time course (mean±SEM): while opto-PV induced pyramidal cell silencing (median decreased in firing rate: 95% at 13Hz (p=0.0009, n=14, Wilcoxon's signed rank test), 100% at 67Hz (p=0.0001), and 100% at 130Hz (p=0.0001), opto-SST only decreased pyramidal cell firing by 67% at 67Hz (p=0.0352, n=10) and by 55% at 130Hz (p=0.0273). **e**, Open field: rotations normalized by the distance travelled in each epoch: a decrease in ipsilateral rotations in opto-stimulated 6-OHDA-*Sst::ChR2* (p=0.0002, n=11, paired t-test) and -*Pv::ChR2* (p=0.0125, n=12) mice, as well as in levodopa (6mg/kg) treated 6-OHDA-wild-type mice (p=2.0x10^{-5}, n=10), but no effect of control opto-stimulation (p=0.2540, n=11) nor DBS (p=0.2456, n=12) in 6-OHDA wild-type mice. **f**, Open field: opto-PV/SST in sham mice does not affect rotational behavioral and locomotor activity (*Pv::ChR2*: n=6, p=0.4311 and p=0.5532; *Sst::ChR2: n*=5, p=0.2537 and p=0.2721, paired t-test). **g**, Cross-maze: opto-PV/SST in sham mice does not lead to preferential turn (*Sst::ChR2: n=5,* ipsilateral turns p=0.3718, straight p=0.5317 and contralateral turns p=0.6062; *Pv::ChR2*: n=6, ipsilateral turns p=0.9416, straight p=0.3861, paired t-test) except for an increase in contralateral turns in *Pv::ChR2* mice (p=0.0272). **e-g**: mean±SEM and individual mice are represented. In all panels, statistical tests are all two-tailed. *: p<0.05; **: p<0.005; ***: p<0.001.

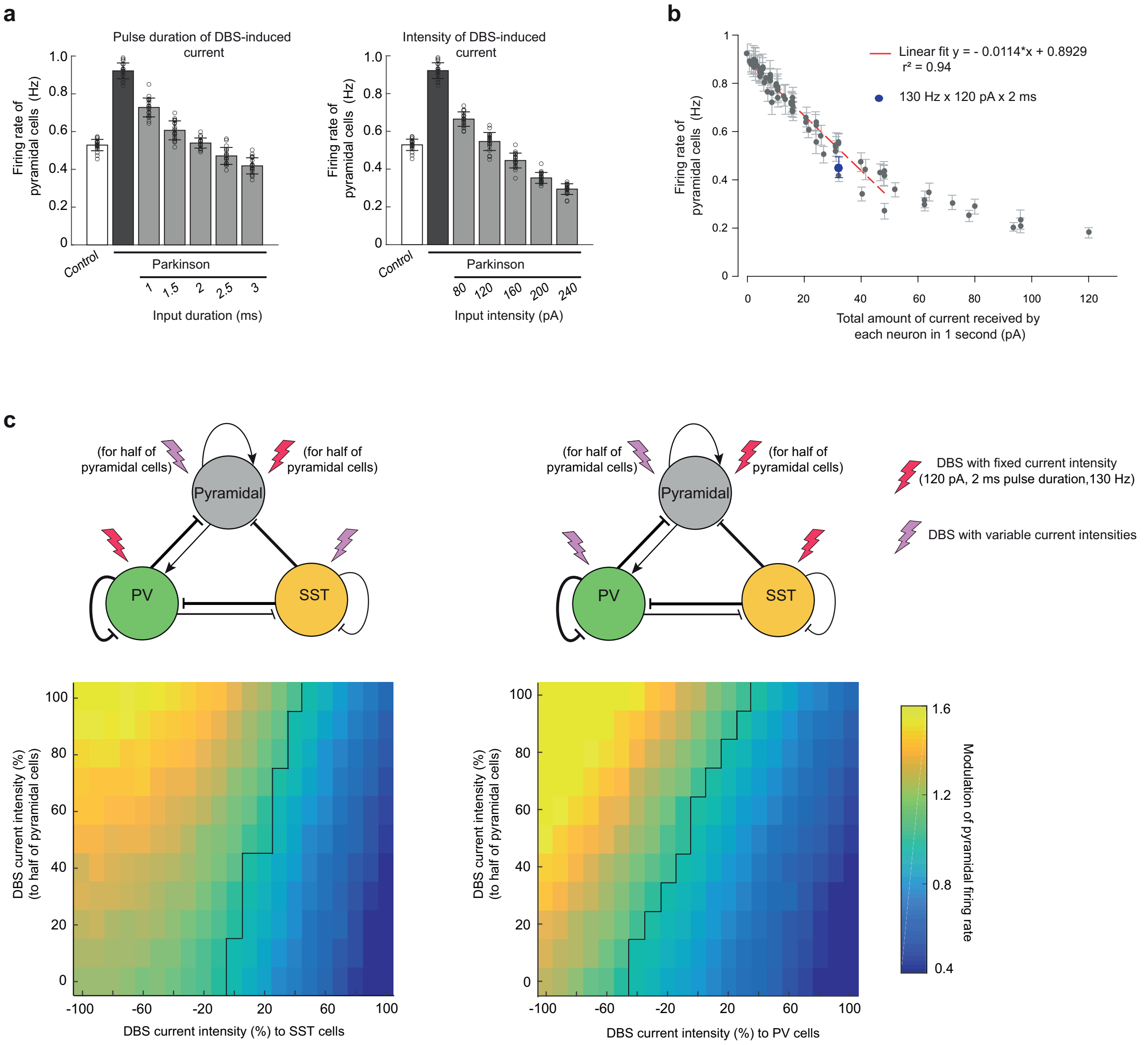

Supplementary Figure 5. Estimating the robustness to changes in DBS parameters.
a, Average firing rates (±SD) of pyramidal cells when varying two parameters of DBS-induced currents for all three populations (pulse duration and intensity). All conditions were significantly different from the parkinsonian condition ($p<0.001$, n=20 independent simulations). **b**, Evolution of pyramidal cell average firing rate (±SD, n=20 independent simulations) as a function of the amount of DBS-induced current injected in the network (total current received by each neuron over one second). All three populations received the same DBS inputs, ranging from 40 to 240pA, with pulse duration lasting from 1 to 3ms, repeated at 13, 67, 130 and 200Hz. A linear fit (red dashed line) was performed, without considering the last eight points corresponding to high intensity currents, showing that the decrease in pyramidal firing rate linearly scales with the total amount of current injected in the network. **c**, Average firing rate of pyramidal cells relative to the parkinsonian condition (n=20 independent simulations) as a function of the percentage of DBS current intensity received by half of pyramidal cells, all SST (left) or PV (right) interneurons. The maximal intensity (100%) corresponds to 120pA (2ms duration). The other half of pyramidal cells receives fixed default DBS-induced current in both cases. Default DBS parameters were used, with a maximal intensity of 120pA. The isocline corresponding to a ratio of 1 is indicated with a black line.

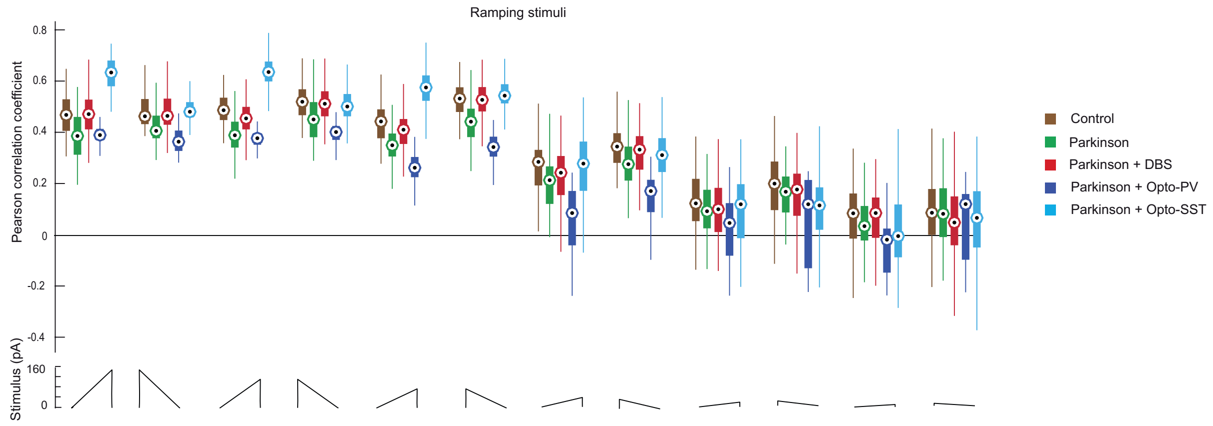

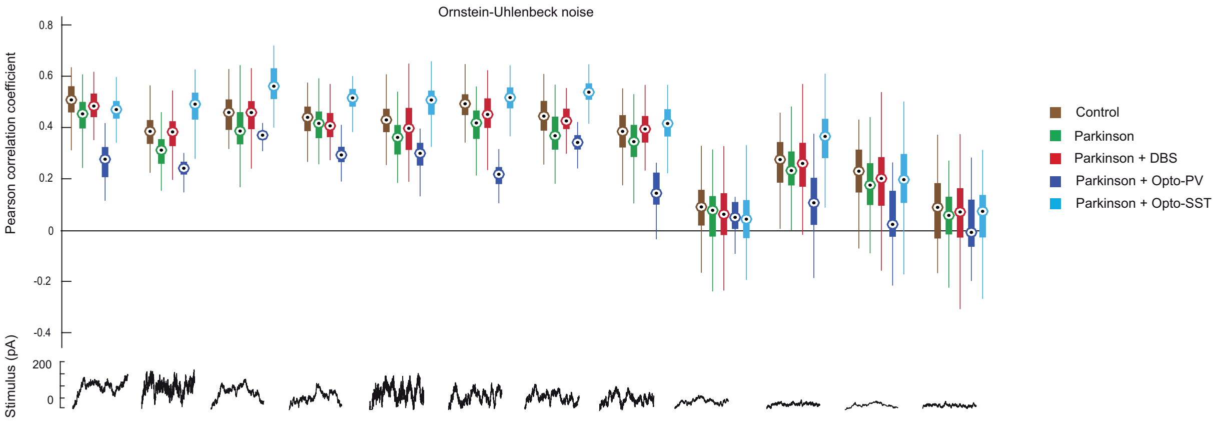

Supplementary Figure 6. Correlations between the network responses and time-varying stimuli.

Pearson correlation coefficients between an input (displayed below) and the moving spike count over all pyramidal cells (with a 10 ms interval), for twelve ramping stimuli (*top*) or Ornstein-Uhlenbeck processes (*bottom*). The line of the box plot indicates the median, the box represents the 25% and 75% quartiles (Q1 and Q3) and the whiskers represents 1.5* the interquartile range outside of the box range, extended to the adjacent value. Data outside the whisker range are not shown, but included in the statistical analysis. Statistical differences are indicated in Fig.S7c (n=100 simulations).

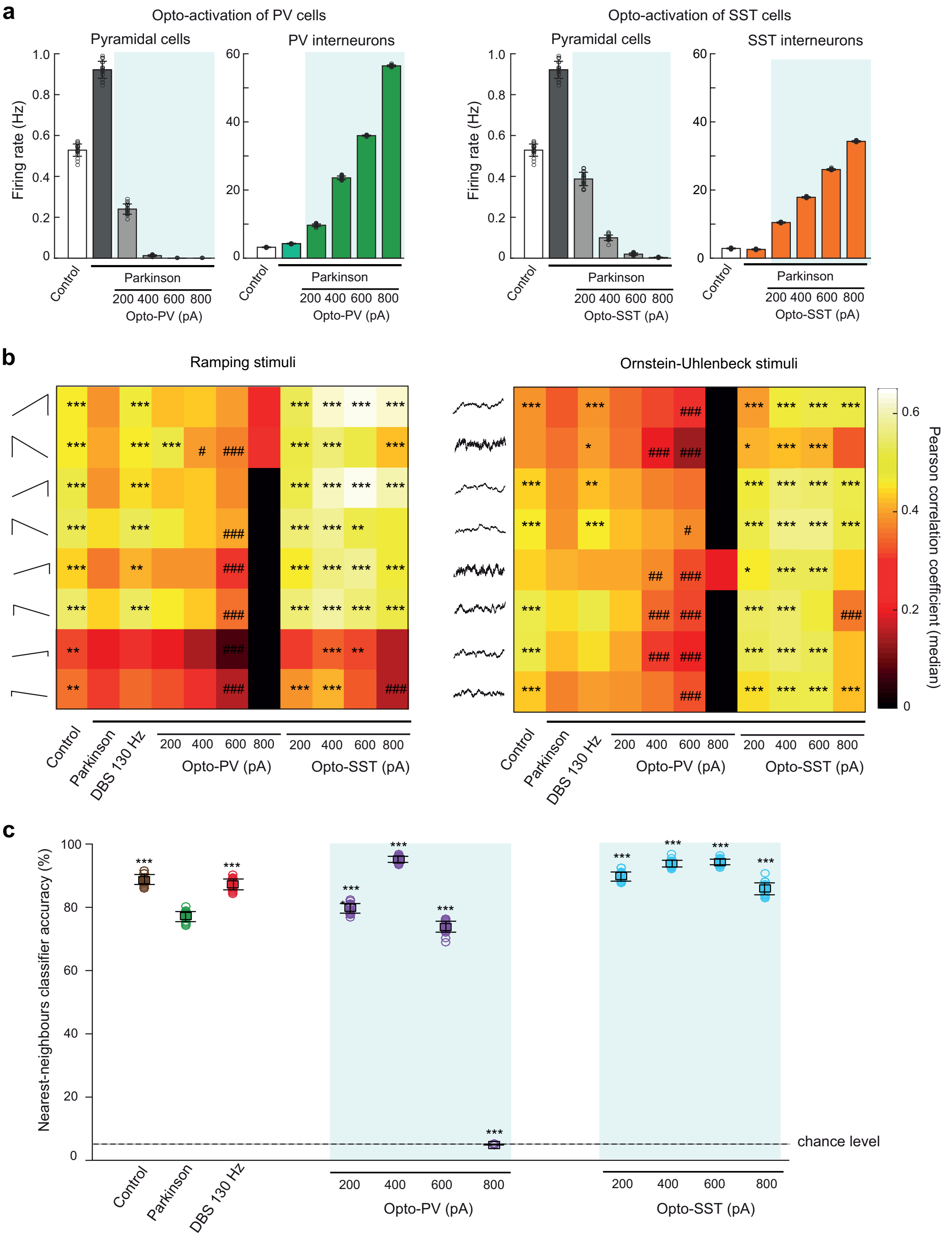

Supplementary Figure 7. Theoretical impact of extreme sparsening of pyramidal cells responses on cortical information processing

a, Population firing rate (mean±SD) across different conditions and for varying intensities of interneuron opto-activation in the absence of additional stimuli. All firing rates are significantly (p<0.001, n=20 independent simulations) different from those observed in the parkinsonian conditions. **b,** Heatmaps of median Pearson correlation coefficients (n=100 independent simulations) for ramping stimuli (left) and stochastic inputs (right). **c,** Accuracy (mean±SD, n=25 independent simulations) of the nearest-neighbours classifier for varying intensities of interneuron opto-activation. Statistical comparisons to the parkinsonian conditions are performed using One-Way ANOVA (or Kruskal-Wallis test for panel b) with Tukey-Kramer *post-hoc* tests, *: p<0.05; **: p<0.01; ***: p<0.001. Statistical tests are all two-tailed. For panels a and c: see Supplementary data 1.

	Pyramidal cells	PV cells	SST cells
E_{leak} (mV)	- 60		
E_{exc} and E_{inh} (mV)	0 and - 80		
τ_{exc} and τ_{inh} (ms)	3 and 5		
σ (mV)	5		
V_{reset} (mV)	- 60		
g_{leak} (nS)	6	5	5
C (pF)	180	80	80
V_{thres} (mV)	-49 (or – 48)	-52	-53
Δ_{thres} (mV)	1	1	5
a (nS)	4	0	4
b (pA)	100	0	90
τ_w (ms)	100	15	40
I_{ext} (pA)	100	50	25

Supplementary Table 1. Intrinsic electrophysiological parameters for pyramidal, PV and SST cells.

Types of input		
Constant	40, 50, 60 and 70 pA	
Ramping	1. From 0 to 160 pA	2. From 160 to 0 pA
	3. From 0 to 120 pA	4. From 120 to 0 pA
	5. From 0 to 80 pA	6. From 80 to 0 pA
	7. From 0 to 40 pA	8. From 40 to 0 pA
	9. From 0 to 20 pA	10. From 20 to 0 pA
	11. From 0 to 10 pA	12. From 10 to 0 pA
Ornstein-Uhlenbeck noise	1. μ =200 pA, σ = 5 pA.ms$^{-0.5}$, τ = 100 ms	
	2. μ = 120 pA, σ = 14 pA.ms$^{-0.5}$, τ = 10 ms	
	3. μ =100 pA, σ = 4 pA.ms$^{-0.5}$, τ = 100 ms	
$dx_t = \frac{(\mu - x_t)}{\tau} dt + \sigma\, dW_t$	4. μ =100 pA, σ = 4 pA.ms$^{-0.5}$, τ = 100 ms	
	5. μ =100 pA, σ = 16 pA.ms$^{-0.5}$, τ = 10 ms	
with $x_0 = 0$	6. μ =80 pA, σ = 8 pA.ms$^{-0.5}$, τ = 20 ms	
	7. μ =60 pA, σ = 6 pA.ms$^{-0.5}$, τ = 20 ms	
	8. μ = 50 pA, σ = 7 pA.ms$^{-0.5}$, τ = 20 ms	
	9. μ = 40 pA, σ = 2 pA.ms$^{-0.5}$, τ = 100 ms	
	10. μ = 20 pA, σ = 2 pA.ms$^{-0.5}$, τ = 10 ms	
	11. μ = 20 pA, σ = 1 pA.ms$^{-0.5}$, τ = 100 ms	
	12. μ = 15 pA, σ = 2 pA.ms$^{-0.5}$, τ = 10 ms	

Supplementary Table 2. Stimuli injected to a subset of pyramidal cells.

All stimuli last for 500 ms.

Algorithms	Functions and Parameters
Nearest centroid classifier	Use of the Euclidean distance<br>No threshold for shrinking centroids
Multinomial logistic regression	Use of the cross-entropy loss for the decision function<br>Using the Newton-cg solver (with L2 regularization)<br>Penalty of the error term: C=1 (default parameter)<br>Tolerance: 0.00001 (default parameter)<br>Maximal number of iterations: 300
Linear discriminant analysis	Using singular value decomposition<br>No priors given, no given knowledge of the number of components for dimensionality reduction<br>Tolerance: 0.0001 (default parameter)
Support vector machines	Use of a “one-against-one” approach for the decision function<br>Using a linear kernel, with a shrinking method<br>Penalty of the error term: C=1 (default parameter)<br>Tolerance: 0.0001 (default parameter)<br>No limit on the number of iterations

Supplementary Table 3. Functions and parameters used for supervised-learning algorithms.
