# Supplementary material for: Deep brain stimulation-guided optogenetic rescue of parkinsonian symptoms
Source: Nat Commun. 2020 May 13;11:2388. doi: 10.1038/s41467-020-16046-6 (PMC7220902; doi:10.1038/s41467-020-16046-6)
Supplement: Supplementary file 4 — Description of Additional Supplementary Files [file 41467_2020_16046_MOESM4_ESM.pdf]

Description of Additional Supplementary Files

File Name: Supplementary Data 1
Description: Detailed statistics (*p* values) for Figures 6 and 7
