# Supplementary material for: Deep brain stimulation-guided optogenetic rescue of parkinsonian symptoms
Source: Nat Commun. 2020 May 13;11:2388. doi: 10.1038/s41467-020-16046-6 (PMC7220902; doi:10.1038/s41467-020-16046-6)
Supplement: Supplementary file 5 — Supplementary Data 1 [file 41467_2020_16046_MOESM5_ESM.pdf]

Supplementary Data 1

Deep brain stimulation-guided optogenetic rescue of parkinsonian symptoms

Valverde *et al.*

Statistics for the computational modeling (*p* values)

Firing rate modulation

One-Way ANOVA followed by Tukey-Kramer *post-hoc* multiple comparison test

Statistics for Figure 6a

Comparison to the parkinsonian condition	Control	DBS 18 Hz	DBS 67Hz	DBS 130 Hz	DBS 180 Hz
Pyramidal neurons	2.07 10^{-8}	2.07 10^{-8}	2.07 10^{-8}	2.07 10^{-8}	2.07 10^{-8}
PV neurons	2.07 10^{-8}	0.0814	2.07 10^{-8}	2.07 10^{-8}	2.07 10^{-8}
SST neurons	3.89 10^{-6}	2.07 10^{-8}	2.07 10^{-8}	2.07 10^{-8}	2.07 10^{-8}

Statistics for Figure 6d

Comparison to the parkinsonian condition	Control	Opto-SST 600 pA. 13 Hz	Opto-SST 600 pA. 67 Hz	Opto-SST 600 pA. 130 Hz	Opto-PV 600 pA. 13 Hz	Opto-PV 600 pA. 67 Hz	Opto-PV 600 pA. 130 Hz
Pyramidal neurons	5.99 10^{-8}	5.99 10^{-8}	5.99 10^{-8}	5.99 10^{-8}	5.99 10^{-8}	5.99 10^{-8}	5.99 10^{-8}
PV neurons	5.99 10^{-8}	5.99 10^{-8}	5.99 10^{-8}	5.99 10^{-8}	5.99 10^{-8}	5.99 10^{-8}	5.99 10^{-8}
SST neurons	5.99 10^{-8}	5.99 10^{-8}	5.99 10^{-8}	5.99 10^{-8}	5.99 10^{-8}	5.99 10^{-8}	5.99 10^{-8}

Correlation coefficients

Kruskal-Wallis followed by Tukey-Kramer *post-hoc* multiple comparison test

Statistics for Figure 7c and Supplementary Figure S7c
Ramping stimuli:

Comparison to PD*	*Control*	*DBS 130 Hz*	*Opto-PV 200 pA*	*Opto-PV 400 pA*	*Opto-PV 600 pA*	*Opto-PV 800 pA*	*Opto-SST 200 pA*	*Opto-SST 400 pA*	*Opto-SST 600 pA*	*Opto-SST 800 pA*
1	1.39 10^{-5}	1.15 10^{-5}	0.348	0.642	0.830	0.985	1.72 10^{-7}	1.72 10^{-7}	1.72 10^{-7}	1.72 10^{-7}
2	1.24 10^{-6}	1.77 10^{-6}	1.75 10^{-7}	0.041	4.41 10^{-5}	0.981	1.72 10^{-7}	1.72 10^{-7}	1.72 10^{-7}	0.999
3	1.27 10^{-7}	1.44 10^{-4}	0.3001	0.387	0.3482	NaN	1.27 10^{-7}	1.27 10^{-7}	1.27 10^{-7}	1.27 10^{-7}
4	1.95 10^{-7}	1.70 10^{-5}	0.4276	0.318	9.84 10^{-7}	NaN	1.27 10^{-7}	1.27 10^{-7}	0.0012	0.856
5	3.58 10^{-7}	0.0037	0.6138	0.905	8.75 10^{-5}	NaN	1.27 10^{-7}	1.27 10^{-7}	1.27 10^{-7}	1.27 10^{-7}
6	1.27 10^{-7}	1.27 10^{-7}	0.6564	0.961	4.08 10^{-7}	NaN	1.27 10^{-7}	1.27 10^{-7}	1.27 10^{-7}	2.60 10^{-4}
7	0.0062	0.721	0.975	0.290	1.11 10^{-4}	NaN	0.0020	1.27 10^{-7}	0.0098	0.294
8	0.0014	0.243	0.979	0.246	2.53 10^{-7}	NaN	1.29 10^{-7}	1.27 10^{-7}	0.938	1.93 10^{-7}
9	0.317	0.995			0.692				0.991	
10	0.401	1.00			0.205				0.017	
11	0.365	0.607			0.287				0.306	
12	0.995	0.603			0.925				0.660	

*: PD: Parkinsonian condition

Statistics for Figure 7c and Supplementary Figure S7c
Ornstein-Uhlenbeck stimuli:

Comparison to PD *	*Control*	*DBS 130 Hz*	*Opto-PV 200 pA*	*Opto-PV 400 pA*	*Opto-PV 600 pA*	*Opto-PV 800 pA*	*Opto-SST 200 pA*	*Opto-SST 400 pA*	*Opto-SST 600 pA*	*Opto-SST 800 pA*
1	6.22 10^{-6}	4.12 10^{-5}	0.593	0.643	1.17 10^{-5}	NaN	1.40 10^{-6}	1.27 10^{-7}	1.27 10^{-7}	1.27 10^{-7}
2	0.214	0.0205	1.00	1.27 10^{-7}	1.27 10^{-7}	NaN	0.0115	1.47 10^{-5}	1.05 10^{-4}	0.970
3	8.53 10^{-6}	0.0020	0.910	0.6053	0.0597	NaN	1.27 10^{-7}	1.27 10^{-7}	1.27 10^{-7}	1.27 10^{-7}
4	1.66 10^{-4}	1.77 10^{-4}	0.748	1.00	0.0165	NaN	1.27 10^{-7}	1.27 10^{-7}	1.27 10^{-7}	1.27 10^{-7}
5	0.6569	1.00	0.894	0.0020	1.72 10^{-7}	0.0954	0.0232	1.72 10^{-7}	1.72 10^{-7}	0.141
6	2.71 10^{-4}	0.0675	0.944	1.27 10^{-7}	1.27 10^{-7}	NaN	1.65 10^{-6}	1.58 10^{-7}	0.799	1.27 10^{-7}
7	2.47 10^{-5}	0.233	0.981	1.27 10^{-7}	1.27 10^{-7}	NaN	7.44 10^{-7}	1.27 10^{-7}	1.28 10^{-7}	1.00
8	2.86 10^{-5}	0.093	0.973	0.801	2.42 10^{-4}	NaN	2.33 10^{-7}	1.27 10^{-7}	1.27 10^{-7}	1.81 10^{-7}
9	0.481	0.645			1.06 10^{-6}				1.35 10^{-8}	
10	0.810	1.00			0.366				1.00	
11	0.055	0.778			6.33 10^{-5}				0.833	
12	0.444	0.998			1.00				0.872	

*: PD: Parkinsonian condition

Classifier accuracy

One-Way ANOVA followed by Tukey-Kramer *post-hoc* multiple comparison test

Statistics for Figure 7d

Comparison to the parkinsonian condition	*Control*	*DBS 130 Hz*	*Opto-PV 600 pA. 67 Hz*	*Opto-SST 600 pA. 67 Hz*
Nearest centroid classifier	9.92 10^{-9}	9.92 10^{-9}	1.01 10^{-8}	9.92 10^{-9}
Multinomial logistic regression	9.92 10^{-9}	9.92 10^{-9}	9.92 10^{-9}	9.92 10^{-9}
Linear Discriminant analysis	9.92 10^{-9}	9.92 10^{-9}	9.92 10^{-9}	9.92 10^{-9}
Support vector machines	9.92 10^{-9}	9.92 10^{-9}	9.92 10^{-9}	9.92 10^{-9}
